# Beyond the *p* -value: Pitfalls in Reporting and Applying Statistical Tests in Sports Science

**DOI:** 10.1055/a-2917-0363

**Published:** 2026-08-05

**Authors:** Roberto C.M. Leite, Rhaí A. Arriel, Rodney Paixão, Leandro Sant’Ana, Moacir Marocolo

**Affiliations:** 1 Integrated Laboratory of Physiology and Performance (LABIFID)Department of Biophysics and Physiology 28113 Federal University of Juiz de ForaJuiz de ForaMinas GeraisBrazil

**Keywords:** statistical analysis, research methodology, methodological rigor, assumption testing, scientific rigor, parametric tests

## Abstract

Statistical inference underpins the credibility of sports science research, yet concerns remain regarding the rigor of statistical reporting and analysis. This study assessed the adequacy of statistical practices in sports science research according to established methodological guidelines. A total of 167 original studies published in 2020 across 15 sports science journals indexed in the Journal Citation Reports (Q1–Q3) were systematically reviewed. Up to 50 studies per journal were randomly selected, and data regarding study characteristics and statistical methods were extracted. Statistical adequacy was classified according to predefined criteria involving reporting omissions and/or statistical misapplications. Overall, 87.4% of studies presented at least one inadequacy. The most frequent reporting omissions involved failure to report assumptions such as normality, homogeneity of variances, and sphericity when required. Common statistical misapplications included the use of inappropriate dependent variables and incorrect treatment of independent observations as dependent measures. Deficiencies were identified across all journal quartiles, with no association between adequacy and journal ranking ( *p* =0.586). These findings raise concerns regarding validity, reproducibility, and practical interpretation in sports science research, reinforcing the need for greater statistical literacy, stricter reporting standards, and improved peer review practices.

## Introduction

Statistical analysis plays a central role in sports science research, supporting the investigation of complex interactions among physiological, biomechanical, and psychological factors that influence athletic performance, injury risk, and recovery outcomes. 1​ Researchers frequently employ a wide range of statistical procedures, including descriptive, inferential, and multivariate analyses to evaluate the effects of interventions, monitor adaptations, and validate theoretical frameworks. The results of these analyses directly inform evidence-based practices aimed at optimizing training, enhancing performance, and reducing injury risk in both elite and recreational athletic populations. 2​ Given the high-stakes nature of competitive sports and the emphasis on marginal gains, the accuracy and validity of statistical findings are essential for translating research into actionable strategies.

The adequate application of statistical tests becomes especially critical when comparing interventions or evaluating performance-enhancing techniques. Selecting the correct statistical procedure requires the careful consideration of study design, data distribution, sample size, and the underlying assumptions of the chosen tests. 3​ ​4​ For example, employing parametric methods such as *t* -tests and analysis of variance (ANOVA) without first verifying assumptions of normality, homogeneity of variance, or sphericity can lead to erroneous interpretations. 4​ These methodological oversights risk generating false positives or masking true effects, which in turn may propagate ineffective or even detrimental practices within applied settings. Consequently, methodological rigor and transparent and comprehensive statistical reporting are indispensable for the advancement of credible, reproducible, and impactful sports science research. 5​

Despite the recognized importance of statistical integrity, there is growing concern regarding the prevalence of reporting omissions and statistical misapplications in the published literature. 6​ 7​ ​8​ In particular, high-impact sports science journals—often perceived as authoritative sources—may inadvertently contribute to the dissemination of flawed findings when these issues go undetected during peer review. Such instances not only undermine the validity of individual studies but may also compromise the scientific credibility of the journals themselves and the broader field, 9​ particularly when incomplete reporting of statistical procedures prevents readers and reviewers from properly assessing the adequacy of the statistical analyses and identifying potential methodological flaws. The propagation of statistically unsound conclusions can mislead future research efforts, misinform practitioners, and, ultimately, impair athlete care and performance optimization.

In light of these concerns, the present study aimed to critically evaluate the adequacy of statistical reporting and analysis in studies published in leading sports science journals, based on established methodological guidelines and best practices. We hypothesized that reporting omissions and statistical misapplications would be widespread and could significantly influence the validity of published findings. By systematically reviewing the statistical methodologies employed in a representative sample of studies, this study sought to identify recurrent errors, highlight areas requiring improvement, and encourage a stronger culture of statistical rigor within the field. Strengthening methodological standards is essential to ensure that the evidence base underpinning sports science remains robust, reliable, and truly beneficial to both the advancement of knowledge and its practical applications.

## Materials and methods

### Study design

This retrospective, descriptive, exploratory study employed a bibliographic survey.

### Selection of scientific journals

Sports science journals were identified from the 2020 Journal Citation Reports (JCR). Journals were selected based on two main criteria: impact factor ranking and accessibility of published studies. To be eligible, journals were required to: (a) belong to the sports science domain; (b) publish original research involving human physical performance; (c) offer accessible archives through databases or open-access platforms; and (d) regularly publish original research studies. Journals were excluded if they primarily published narrative reviews, systematic reviews, meta-analyses, editorials, opinion pieces, animal studies, or studies unrelated to human physical performance. Journals included in the analysis were classified into quartiles based on their 2020 JCRs impact factor rankings, where Q1 (1st quartile) represents the highest-ranked journals and Q4 (4th quartile) the lowest-ranked journals in the sports science category. The year 2020 was selected because the development of this project began in 2021, making it the most recent complete publication year prior to the start of the study. This ensured that all journal issues had been fully published.

### Study selection

From each selected journal, complete issues published in 2020 were randomly sampled. From these issues, up to 50 studies per journal were randomly selected. Studies were accessed through the journals’ official websites or via institutional databases and academic platforms (e.g., PubMed, ScienceDirect, SpringerLink, Wiley Online Library, and ResearchGate), ensuring comprehensive and standardized data retrieval.

Two independent reviewers screened studies for eligibility, and any discrepancies were resolved through consultation with a third reviewer. Initially, studies were screened based on titles and abstracts according to the predefined eligibility criteria. Subsequently, full-text studies were assessed for final inclusion. Studies were included if they met the following criteria: (1) original research studies published in 2020; (2) studies involving physical activity, exercise, or human physical performance; (3) studies clearly describing the statistical methods used; and (4) studies employing statistical tests for group comparisons (e.g., between or within-subject designs). Studies were excluded if they met any of the following criteria: (1) narrative reviews, systematic reviews, meta-analyses, editorials, and opinion articles; (2) studies with predefined or fixed statistical procedures (e.g., data derived from pre-calculated indices or algorithms with no statistical testing performed by the authors), or studies that did not report or apply any statistical analysis; (3) studies involving animal models or cadaveric specimens; (4) studies not available in the full-text; and (5) studies exclusively using associations analyses (e.g., correlation or regression), or that combined association and group-comparison analyses in a way that prevented the isolated evaluation of comparison-based statistical methods.

In this context, association analyses refer to the evaluation of relationships between two or more variables (e.g., using Pearson's correlation or linear regression), whereas comparison analyses refer to the assessment of differences between or within groups (e.g., *t* -tests or ANOVA). This distinction was adopted because, although both approaches rely on statistical assumptions, they address fundamentally different research questions.

### Data extraction

Data were extracted from each study regarding the journal name, impact factor, quartile, year, issue, and title; sample size; type of observations (paired or independent); variables (categorical: ordinal or nominal and numerical: discrete or continuous); and general statistical methodology (statistical tests and their assumptions).

### Evaluation of statistical reporting and analysis

The adequacy of statistical reporting and analysis in the included studies was specifically evaluated using predefined criteria derived from guidelines proposed by Motulsky, 10​ ​11​ Mangiafico, 4​ and Mahrholz and Kuepper-Tetzel, 3​ as listed in Table 1 . A study was considered adequate if none of these criteria were identified, and inadequate if at least one was present. Studies classified as inadequate were further categorized according to the type of issue identified: (1) reporting omissions, (2) statistical misapplications, or both. Reporting omissions referred to situations in which the essential statistical information was missing or insufficiently reported. Statistical misapplications were defined as the incorrect use of statistical procedures, such as violations of statistical test assumptions that could be identified based on the information reported in the study. Studies with alternative robustness strategies reported, such as inspecting residuals to evaluate normality, were taken into account in the analysis. In studies employing more than one statistical test for comparison, each test was examined and evaluated individually. The study was classified as inadequate if any of the tests met at least one of the predefined criteria.

**Table:** **Table 1** Criteria used to evaluate the adequacy of statistical reporting and analysis in the included studies

Criteria	Explanation	Category
Failure to report the assessment of test assumptions	The authors did not report whether the assumptions of the statistical test were checked.	Reporting omissions
Violation of statistical assumptions	One or more assumptions of the statistical test were violated based on the information reported in the article (e.g., The authors used a one-way ANOVA, but the assumption of independence of observations was violated because the same participants contributed data to more than one group, leading to correlated observations)	Statistical misapplications
Statistical test not reported	The authors did not mention which statistical test was used to analyze the data (e.g., they mentioned that a comparison between two groups had been made but did not specify the statistical test performed).	Reporting omissions
Incomplete specification of the statistical test	The authors did not specify which statistical test was applied (e.g., they described that a *t* -test was performed but did not specify whether it was a paired or unpaired *t* -test).	Reporting omissions

Abbreviation: ANOVA, analysis of variance.

Two independent researchers systematically reviewed each study and classified it as adequate or inadequate based on the predefined criteria listed in Table 1 . In cases where there was a disagreement between the two reviewers, a third expert reviewer was consulted. This third reviewer re-evaluated the study and considered the assessments provided by the initial reviewers. The final classification was determined by consensus among the three evaluators.

### Statistical analysis

All statistical analyses were conducted using IBM SPSS Statistics (Version 23; IBM Corp., Armonk, NY, USA). The Shapiro–Wilk test was used to assess the normality of the numerical data distributions in order to determine the most adequate descriptive statistics. Data from variables that did not meet the assumption of normality were summarized using medians and interquartile ranges (IQR), while categorical variables were described using absolute and relative frequencies. To examine the relationship between the adequacy of statistical reporting and analysis (adequate vs. inadequate) and the quartile classification of the journal (Q1 to Q3), Fisher’s exact test was applied because some expected cell counts were below five. When significant associations were detected, pairwise comparisons were conducted using the *z* -test, with *p* -values adjusted using the Bonferroni correction to control for multiple comparisons. 12​ Additionally, adjusted residuals were analyzed to identify cells contributing significantly to the association; residuals with values below –1.96 or above 1.96 were considered statistically significant. 13​ Finally, inter-reviewer agreement on the adequacy of statistical reporting and analysis was assessed using Cohen’s kappa coefficient, interpreted as follows: poor (<0.00), slight (0.00–0.20), fair (0.21–0.40), moderate (0.41–0.60), substantial (0.61–0.80), and perfect (0.81–1.00). 14​ The level of statistical significance was set at *p* <0.05 for all analyses.

## Results

**Fig. 1:**
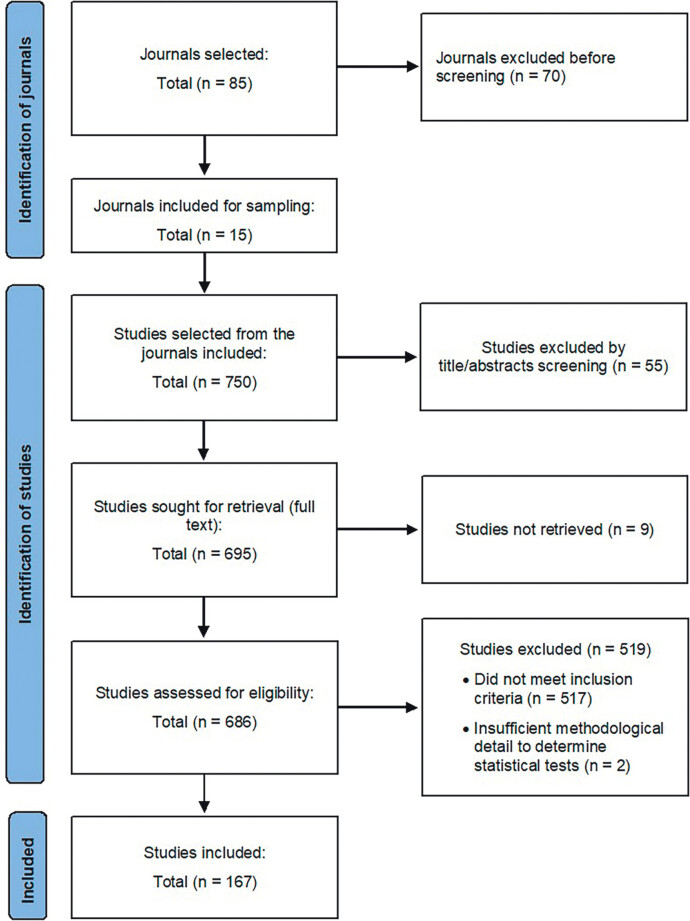
Flowchart—Steps of journal and study selection.

Initially, 85 sports science journals were identified; of which, 15 journals met the inclusion criteria and were included in the analysis. Of these journals, nine journals were classified as Q1, five journals as Q2, and one journal as Q3. A total of 750 studies were identified across 15 journals, covering a mean of 6 issues per journal (range: 2–21). Following title and abstract screening according to the predefined eligibility criteria, 55 records were excluded. The remaining 695 articles were assessed for full-text eligibility. Of these, nine could not be retrieved, two provided insufficient methodological details to determine the statistical tests used, and 517 were excluded for not meeting the inclusion criteria. Thus, 167 studies were included in the final analysis. In some journals, fewer studies met the inclusion criteria within the sampled issues, resulting in lower final counts. The journal and study selection process is shown in Fig. 1 . The characteristics of the included journals and the number of studies included per journal are presented in Table 2 .

**Table:** **Table 2** Characteristics of selected journals

Journals	Impact factor	Quartile rankings	Studies included
British Journal of Sports Medicine	13.8	1	1
*The American Journal of Sports Medicine*	6.2	1	5
*Medicine & Science in Sports & Exercise*	5.41	1	20
*Journal of Orthopaedic & Sports Physical Therapy*	4.75	1	1
*Research in Sports Medicine*	4.67	1	12
*International Journal of Sport Nutrition and Exercise Metabolism*	4.6	1	5
*Journal of Science and Medicine in Sport*	4.32	1	6
*Scandinavian Journal of Medicine & Science in Sports*	4.22	1	6
*European Journal of Sport Science*	4.05	1	18
*Journal of Strength and Conditioning Research*	3.78	2	21
*Journal of Sports Sciences*	3.34	2	6
*International Journal of Sports Medicine*	3.12	2	14
*European Journal of Applied Physiology*	3.08	2	19
*Journal of Athletic Training*	2.86	2	20
*Physical Therapy in Sport*	2.37	3	13

Note: The number of included studies per journal varied according to the number of eligible articles identified within the sampled issues. The number of studies screened per journal is reported to provide the corresponding denominators. Journals with low counts (e.g., *n* =1) reflect a limited number of eligible studies meeting the inclusion criteria rather than restricted sampling.

The included studies presented the sample size with a median of 25 (IQR: 38), ranging from a minimum of 5 to a maximum of 17,525 participants. Regarding the types of observations, 66 studies (39.5%) analyzed dependent data, 80 studies (47.9%) analyzed independent data, and 21 studies (12.6%) analyzed both types. For dependent variables, categorical outcomes were reported in 10 studies (6.0%) with nominal data, 46 studies (27.5%) with ordinal data, and 3 studies (1.8%) with both; however, 108 studies (64.7%) did not use categorical variables. Concerning numerical outcomes, 115 studies (68.9%) used continuous data, none reported discrete data, 49 studies (29.3%) included both, and 3 studies (1.8%) did not use numerical variables. The types of statistical tests employed across the studies are summarized in Table 3 . The most commonly used tests were repeated-measures two-way ANOVA, two-sample *t* -test, and paired *t* -test.

**Table:** **Table 3** Statistical tests used in the studies

Statistical test	Number of studies
Repeated-measures two-way ANOVA	49
Two-sample *t* -test	41
Paired *t* -test	39
Repeated-measures one-way ANOVA	28
One-way ANOVA	27
Mann–Whitney *U-* test	20
ANCOVA	14
Two-way ANOVA mixed model	12
Wilcoxon signed-rank test	11
Two-way ANOVA	11
Repeated-measures three-way ANOVA	10
Kruskal–Wallis test	8
Friedman test	4
Three-way ANOVA mixed model	3
Linear mixed model for repeated measures	3
MANOVA	2
Repeated measures MANOVA	2
Three-way ANOVA	1
Four-way ANOVA	1
Nonparametric two-way ANOVA type test	1
Negative binomial regression model	1
Chi-square test	1
Generalized estimating equations model	1
Repeated-measures four-way ANOVA	1

Abbreviations: ANCOVA, analysis of Covariance; ANOVA, analysis of variance; MANOVA, multivariate analysis of variance.

Note: Counts represent the number of studies that used each test at least once. Studies may be counted more than once if they employed several tests.

**Fig. 2:**
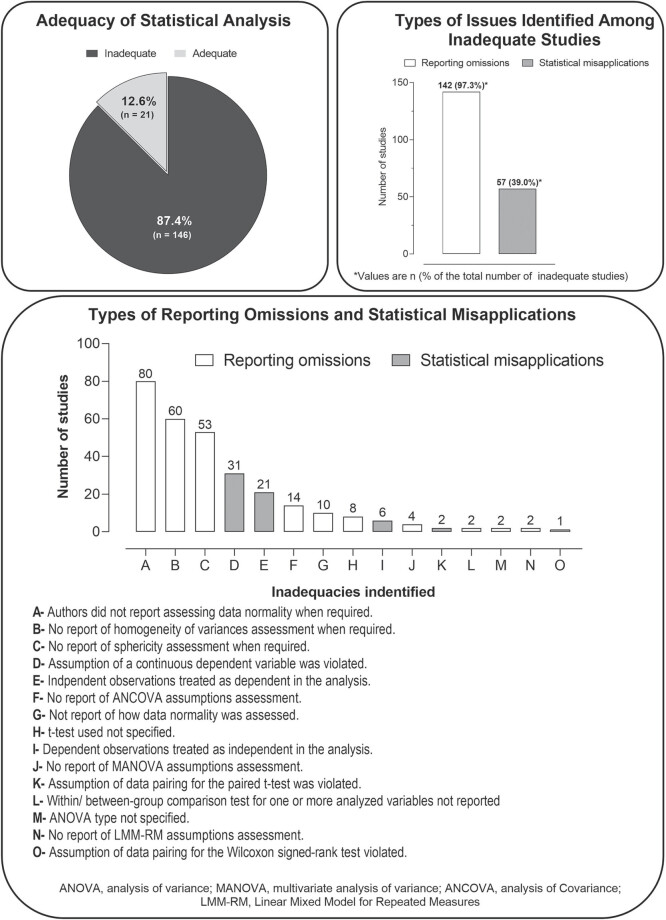
Adequacy of statistical reporting and analysis and types of inadequacies identified in the included studies.

Among the 167 studies analyzed, 146 studies (87.4%) exhibited inadequate statistical reporting or analysis in at least one of the tests applied, as illustrated in Fig. 2 . Of the 146 studies, 142 studies (97.3%) showed reporting omissions and 57 studies (39.0%) showed statistical misapplications. Notably, 53 studies (36.3%) had both issues, 89 studies (61.0%) only reporting omissions, and 4 studies (2.8%) only statistical misapplications. The most frequent reporting omissions concerned assessments of data normality, homogeneity of variances, and sphericity when required, while the most common statistical misapplications involved violating the assumption of a continuous dependent variable and treating independent observations as dependent in the analysis. Furthermore, none of the studies employing ANCOVA or MANOVA reported that their assumptions had been checked. Inter-reviewer agreement on the adequacy of statistical reporting and analysis was moderate ( *κ* =0.461, 95% CI 0.265–0.657, and *p* <0.01).

Studies published in Q1 journals exhibited a slightly higher frequency of inadequacies (90.5%), followed by Q2 (85.0%) and Q3 (84.6%). However, Fisher’s exact test (Freeman–Halton extension) showed no significant association between adequacy of statistical reporting and analysis (adequate vs. inadequate) and quartile rankings (two-sided *p* =0.586; Table 4 ). Among the articles classified as inadequate, reporting omissions were extremely frequent, affecting 65 of 67 studies in Q1 (97.0%), 66 of 68 studies in Q2 (97.1%), and 11 of 11 studies in Q3 (100%). Statistical misapplications were observed in 26 of 67 studies in Q1 (38.8%), 30 of 68 studies in Q2 (44.1%), and 1 of 11 studies in Q3 (9.1%). Some studies presented both issues, so counts are not mutually exclusive.

## Discussion

The present study sets out to evaluate the adequacy of statistical reporting and analysis in original research articles published in leading sports science journals, with the expectation that reporting omissions and statistical misapplications would be common. The findings confirmed this hypothesis, revealing that nearly 9 of 10 studies (87.4%) failed to fully adhere to established methodological guidelines. The most frequent shortcomings were reporting omissions, particularly the failure to report assumption testing for normality, homogeneity of variances, and sphericity in repeated-measures designs when required. The most common statistical misapplications, although less prevalent, included violating the assumption of a continuous dependent variable and treating independent observations as dependent in the analysis. Importantly, these inadequacies were pervasive across all journal quartiles, indicating that methodological rigor is not guaranteed even in higher-ranked outlets. Collectively, these results highlight a systemic issue within sports science publishing and emphasize the urgent need for improved statistical literacy, stricter reporting standards, and enhanced peer-review practices to strengthen the reliability and reproducibility of research in the field.

### Inadequacies in statistical reporting and analysis

The most common inadequacies identified were omissions in reporting assumption testing, particularly for normality, homogeneity of variances, and sphericity in repeated-measures designs. In addition to these reporting issues, several studies also exhibited statistical misapplications, most notably the use of non-continuous dependent variables in tests requiring a continuous dependent variable and the incorrect treatment of independent observations as dependent in the analysis. These shortcomings mirror previous concerns raised in broader biomedical and behavioral sciences, where insufficient reporting and inadequate verification of assumptions remain pervasive obstacles to methodological rigor. 3​ ​10​ The consistent neglect of these requirements suggests not only gaps in statistical literacy but also possible deficiencies in the peer-review process, where such methodological issues appear insufficiently scrutinized.

A striking example is the frequent application of parametric tests without the verification of underlying assumptions. The use of ANOVA and *t* -tests accounted for the majority of analyses, yet their validity critically depends on assumptions that were seldom reported as tested. Similarly, studies applying ANCOVA or MANOVA failed to report all underlying assumptions, despite the complexity and sensitivity of these models to violations. Such practices increase the risk of Type I and Type II errors, thereby undermining the validity of reported effects and limiting the generalizability of findings. 15​

### Journal quartile classification and the adequacy of statistical reporting and analysis

Interestingly, the frequency of statistical inadequacies did not significantly differ across journal quartiles, with Q1 journals—despite their higher impact factors—exhibiting inadequacy rates comparable to Q2 and Q3 outlets. This lack of association suggests that journal prestige and impact factor are not reliable indicators of statistical rigor.

A notable pattern in the results was the extremely high prevalence of reporting omissions across all quartiles. Nearly all studies classified as inadequate failed to report essential aspects of statistical procedures, indicating that insufficient transparency in statistical reporting is a widespread issue rather than one restricted to lower-ranked journals. This pattern reinforces concerns that many published studies provide limited information about the verification of statistical assumptions or other analytical details necessary for proper interpretation and reproducibility. In addition, statistical misapplications were observed in both Q1 and Q2 journals at comparable frequencies, indicating that the incorrect use of statistical procedures is not limited to journals with lower bibliometric rankings. Although the frequency of misapplications was lower in Q3 journals, this pattern should be interpreted cautiously given the small number of studies in this quartile. Together, these findings support previous critiques that bibliometric metrics, such as impact factor, do not necessarily reflect methodological quality or robustness of published evidence, 16​ ​17​ highlighting the importance of rigorous statistical evaluation by editorial boards and peer reviewers across all journal tiers.

### Implications for research transparency and reproducibility

The inadequacies observed raise important concerns about transparency and reproducibility in sports science research. Inadequate reporting of statistical procedures restricts the ability of readers, practitioners, and meta-analysts to critically appraise or replicate findings. Reproducibility crises have been extensively documented across biomedical and psychological sciences, 18​ and the current findings suggest that sports science is equally vulnerable. The implications extend beyond academia, given that applied recommendations in sports performance, clinical exercise physiology, and rehabilitation often derive from such research. Misinterpretations due to poor statistical practice may therefore have direct consequences on evidence-based practice.

### Possible explanations and solutions

Several explanations may account for the high prevalence of inadequate practices. First, limited formal training in statistics among sports science researchers 19​ may lead to over-reliance on conventional tests without a critical evaluation of their assumptions. Second, editorial constraints—such as word limits—might contribute to under-reporting of assumption testing procedures. Finally, peer reviewers may prioritize novelty and practical relevance over methodological rigor, inadvertently allowing flawed analyses to pass unchecked.

**Table:** **Table 4** Counts, expected counts, and adjusted residuals for the adequacy of statistical reporting and analysis (adequate vs. inadequate) by journal quartile rankings (Q1, Q2, and Q3) based on the journal impact factor

		Q1	Q2	Q3	Total
Adequate	Count	7	12	2	21
	Expected count	9.3	10.1	1.6	21
	Adjusted residual	−1.1	0.9	0.3	–
Inadequate	Count	67	68	11	146
	Expected count	64.7	69.9	11.4	146
	Adjusted residual	1.1	−0.9	−0.3	–

Note: Adjusted residual higher than 1.96 or less than − 1.96 means a significant difference between the count and the expected count.

To address these issues, a multifaceted strategy is warranted. First, graduate training programs should emphasize both theoretical understanding and applied competence in statistical methodology, particularly with respect to assumption checking and transparent reporting. In addition, greater involvement of statisticians or researchers with strong methodological expertise throughout all stages of data analysis in research projects may help reduce reporting omissions and statistical misapplications, and improve the overall quality of analytical practices in sports science. At the editorial level, journals could enforce stricter author guidelines requiring explicit documentation of assumption testing and justification of statistical choices, including a dedicated subsection reporting assumption checks (e.g., normality, homogeneity of variances, and sphericity when applicable) or the adoption of statistical reporting checklists to improve transparency and completeness, similar to initiatives adopted in medical research, such as the CONSORT and SAMPL guidelines. 20​ Encouraging the reporting of effect sizes with corresponding uncertainty intervals and the use of more flexible analytical approaches, such as mixed-effects models when assumptions are not met, may further improve the robustness and interpretability of statistical analyses in sports science. Additionally, integrating specialized statistical reviewers into the peer-review process may strengthen the methodological evaluation of submitted manuscripts 6​ and detect potential methodological shortcomings before publication.

### Strengths and limitations

The strengths of this study include its systematic approach, rigorous inclusion criteria, and independent review process with consensus validation, which minimized subjectivity in classifying adequacy. Nonetheless, limitations should be acknowledged. The study was not pre-registered and relied on a retrospective design, which restricted analyses to what was reported in the published studies; thus, some studies may have conducted assumption checks but failed to report them. Moreover, only journals indexed in JCR 2020 were included, which, although representative of the field, excludes emerging or lower-ranked outlets that may present different patterns. Another consideration is that studies were treated as independent despite being nested within journals, which may have slightly influenced uncertainty estimates. Finally, the focus on comparison-based statistical tests excluded correlation and regression approaches, which are also prone to misuse and merit separate investigation.

### Conclusions and perspectives

In summary, this study demonstrates that statistical inadequacies are pervasive in sports science research, transcending journal quartiles and impact factors. The findings highlight an urgent need for improved statistical training, greater transparency in reporting, and enhanced rigor in editorial and peer-review practices. Addressing these shortcomings is critical not only for advancing methodological standards but also for safeguarding the credibility and practical applicability of sports science research.

## Data Availability

The data sets generated and/or analyzed during this study are available in the Open Science Framework (OSF) repository: https://doi.org/10.17605/OSF.IO/79AD8 .
